# Progressive Encephalomyelitis with Rigidity and Myoclonus (PERM) Associated with GlyR Antibody in an APECED Patient

**DOI:** 10.1007/s10875-024-01802-w

**Published:** 2024-09-12

**Authors:** Sebastian Ochoa, Patrick Waters, Eléonore Vieillard, Ariane Soldatos, M Isabel Leite, Michail S Lionakis

**Affiliations:** 1 Laboratory of Clinical Immunology and Microbiology, National Institute of Allergy and Infectious Diseases (NIAID), NIH, Bethesda, MD USA; 2 Department of Pediatrics, Baylor College of Medicine, Houston, TX USA; 3 Nuffield Department of Clinical Neurosciences, University of Oxford, Oxford, UK; 4 National Institute of Neurological Disorders and Stroke, National Institutes of Health, Bethesda, MD USA; 5 Department of Neurology, Oxford University Hospitals NHS Foundation Trust, Oxford, UK

To the editor:

Autoimmune encephalitis (AE) is an infrequent but life-threatening clinical manifestation of inborn errors of immunity (IEI). Autoantibodies to neuronal ion channels, neurotransmitter receptors or associated proteins have been reported in certain patients with IEI and neurological symptoms [1], and their identification has led to targeted and effective therapeutic interventions [2]. Although unexplained neurological syndromes are increasingly recognized in patients with IEI associated with impaired immune tolerance, the underlying causes remain largely unexplored. Autoimmune polyendocrinopathy-candidiasis-ectodermal dystrophy (APECED), also known as Autoimmune polyendocrine syndrome type 1 (APS-1), is an autosomal recessive IEI featuring defective central tolerance, which is most often caused by biallelic deleterious variants in the autoimmune regulator (*AIRE*) gene [3] and characterized by multiple autoimmune manifestations in endocrine and non-endocrine organs. Despite a broad spectrum of autoantibodies observed in APECED patients, AE is not a frequent manifestation in these patients. Here, we describe a patient with APECED who developed neurological symptoms consistent with progressive encephalomyelitis with rigidity and myoclonus (PERM), associated with autoantibodies to glycine α receptor 1 (GlyR). To our knowledge, this is the first report of PERM in APECED.

## Case

The patient is a boy of English and Scottish ancestry with compound heterozygous pathogenic variants in *AIRE* (p.Pro422fs and p.Leu81Pro) and multiple autoimmune manifestations of APECED, including APECED rash, enamel hypoplasia, hypoparathyroidism, adrenal insufficiency, type 1 diabetes, alopecia, and growth hormone and testosterone deficiencies. He was managed only with hormonal replacement (growth hormone, testosterone, hydrocortisone, fludrocortisone and an insulin pump). He did not have a personal history of neurological disease or substance abuse. Family history was negative for neurologic conditions. At age 16, he presented with progressive mild blurred vision and bilateral ptosis, which was variable, possibly worse at the end of the day, as well as intermittent paresthesia and hyperesthesia in the torso and bilateral lower extremities. Over the course of two months, his symptoms worsened, and he developed diplopia, mild dysarthria, occasional mild dysphagia, certain level of tongue stiffness, noticeable when wanted to do rapid movements, mild urinary urgency, constipation, occasional postural dizziness, and brisk involuntary jerky movements triggered by loud sounds and tactile stimuli on the face, neck and upper part of the chest. Neurological examination demonstrated bilateral ptosis, horizontal and vertical nystagmus, limited left eye abduction, neck stiffness, brisk deep reflexes, difficulty with tandem gait and hyperekplexia (see supplemental materials for a detailed neurological examination). There were no neuropsychiatric symptoms (including cognitive impairment, psychosis, or other behavioral changes), involuntary movements, or seizures. Laboratory evaluation showed mild hypocalcemia with otherwise normal electrolytes, renal function, and liver function tests. Immunological evaluation showed normal CD4, CD8, B cell and NK cells; a slightly reduced IgM with otherwise normal IgG and IgA plasma concentration; and normal C3 and C4. Autoantibodies to IFNα, IFNω, IL-17 F, IL-22, GAD65, and 21-hydroxylase were detectable in serum (Table S1). Cerebrospinal fluid (CSF) studies, including protein glucose, cell count, cultures, and evaluation for viral etiologies, were normal. Brain computed tomography (CT) demonstrated basal ganglia calcifications, which is often seen in patients with hypoparathyroidism, including those with APECED. Brain magnetic resonance imaging (MRI) showed only increased T1 hyperintensity corresponding to the areas of calcification seen on CT. Spinal cord MRI was normal (Figure S1). His ocular and bulbar symptoms led to evaluations for myasthenia gravis (MG). Clinically there were no clear features of MG, and autoantibodies to AChR, MUSK and LRP4, by cell-based assay, were negative. A therapeutic trial with pyridostigmine (up to 60 mg twice a day) was advised while waiting test results, which was discontinued after 4 weeks due to absence of clinical improvement.

The patient was evaluated in a multidisciplinary team with experts on myasthenia gravis, congenital myasthenia syndromes and autoimmune neurology of CNS at the John Radcliffe Hospital, Oxford. His disease presentation prompted testing for GlyR autoantibodies in serum and CSF, which were processed in the Oxford Autoimmune Neurology Diagnostic Laboratory at the Nuffield Department of Clinical Neurosciences, University of Oxford. Patient serum (tested at three different timepoints, several weeks apart, for confirmation and reproducibility) and CSF were positive for GlyR [2] (Table 1). The constellation of clinical stiffness or rigidity of mainly ocular, bulbar and neck muscles hyperekplexia, and autonomic symptoms together with GlyR autoantibodies confirmed the diagnosis of PERM, although he did not have myoclonus. To evaluate the specificity of these autoantibodies in the context of APECED, we tested 33 APECED patients without PERM or any other neurological symptoms for GlyR autoantibodies, all of whom were negative. The patient was treated with a 5-day course of intravenous immunoglobulin (IVIG; 2 g/kg), which resulted in significant improvement of his symptoms. Approximately one year later he had a recurrence of ptosis, dysphagia, paresthesia, and tongue stiffness. He was started on monthly immunomodulatory dose IVIG for 6 months (2 g/kg per dose), resulting in significant improvement of his symptoms, with some residual mild ptosis, hyperesthesia and paresthesia on the upper part of the chest and neck, which did affect his normal life.

**Table 1:** Autoantibodies to GlyR in serum and CSF

Sample	Fluorescence score at low dilution*	Fluorescence score at high dilution
Serum (timepoint 1)	4	3
Serum (timepoint 2)	4	3
Serum (timepoint 3**)	3	2
CSF	2.5	1

*HEK 293 cells were transiently transfected with GlyRα1 are exposed to patient serum (at 1:20 and 1:100 dilutions), patient CSF (at 1:2 and 1:20 dilutions) or normal control serum at equivalent dilutions. Autoantibodies to GlyαR1 are detected via immunofluorescence and scored using a semiquantitative score (0 = no binding; 1–2 = low level binding; 2–4 = increasing strength of binding; a score of 1 or higher is considered positive) [2].

**All timepoints preceded the initiation of IVIG.

## Discussion

PERM is a potentially severe autoimmune condition of the CNS, associated with GlyR abs, which can clinically overlap with stiff person syndrome (SPS) but presents with distinct and more severe neurological symptoms. It may present with ocular, facial, bulbar, autonomic, and limb motor features or dysfunction, where muscle rigidity, hyperekplexia and myoclonus are typical, though not always all present at the same time; all reflect an underlying pathology in the brainstem and sometimes spinal cord, although MRI scans are usually reported as normal. This patient brain CT and MRI scan changes are related to his associated autoimmune disease (hypoparathyroidism). Although PERM can occur in isolation, < 10% of patients have concomitant or preceding malignancy (predominantly thymoma or lymphoma) or autoimmune diseases (most commonly thyroiditis, type 1 diabetes, or connective tissue disorders). A study in consecutively enrolled patients with PERM uncovered the presence of autoantibodies to GlyR, a glycine-activated chloride channel which mediates neuronal inhibition and is highly expressed in the brainstem and spinal cord [2]. Although more studies may be required to better understand the pathogenesis of PERM, studies showing GlyR autoantibody-induced internalization of GlyR and complement-mediated neuronal damage suggest that these autoantibodies may disrupt GlyR + inhibitory neurons [2]. Notably, PERM presented later than other APECED autoimmune manifestations, thus it may be a rare, late-onset manifestation of APECED, although it presented early when compared to other PERM cases described (Table S2).

Detection of autoantibodies to GlyR has important diagnostic and therapeutic implications. GlyR autoantibodies are detected in serum in most patients with clinical features of this very rare autoimmune condition, PERM [2] and can help establish the diagnosis. It is important to note that while these antibodies are useful diagnostically, titers do not correlate with disease severity or response to treatment. Furthermore, reports of patients with PERM with detectable GlyR autoantibodies in CSF but not in serum [4] highlight the importance of obtaining CSF in addition to serum samples. In the prospective cohort of 33 patients with PERM and GlyR autoantibodies, most patients experienced objective symptom improvement with plasmapheresis and/or immunomodulatory dose IVIG [2]. Approximately 20% had disease relapses, managed with mycophenolate, azathioprine or regular IVIG, with good clinical responses [2]. Although the presence of anti-GlyR autoantibodies has not been systematically evaluated in patients with APECED or in other patients with IEI, our findings illustrate how early identification can lead to initiation of appropriate treatment before the onset of irreversible neurological sequelae. Our patient was also positive for GAD65 autoantibodies, known to be associated with SPS but found only a minority of patients with PERM [2]. Moreover, the majority of APECED patients carry GAD65 autoantibodies indicating that their presence is not specific for PERM [5]. None of the tested APECED patients without encephalopathy were positive for GlyR autoantibodies, suggesting these are specific for PERM in APECED.

Our report illustrates the importance of testing for autoantibodies to specific neuronal antigens in patients with IEI presenting with unexplained neurological symptoms and further expands the spectrum of autoimmune manifestations that can be observed in patients with APECED.

## Electronic Supplementary Material

Below is the link to the electronic supplementary material.

Supplementary Material 1

## Data Availability

No datasets were generated or analysed during the current study.
