# Supplementary material for: Progressive Encephalomyelitis with Rigidity and Myoclonus (PERM) Associated with GlyR Antibody in an APECED Patient
Source: J Clin Immunol. 2024 Sep 12;45(1):2. doi: 10.1007/s10875-024-01802-w (PMC11393104; doi:10.1007/s10875-024-01802-w)
Supplement: Supplementary file 1 — Supplementary Material 1 [file 10875_2024_1802_MOESM1_ESM.docx]

**Table S1. Laboratory results of our APECED patient.**

| **Test Name** | **Result** | **Reference range** |
| --- | --- | --- |
| CD3+ cells | 2.028 | 0.7 – 4.2 x 10^9^ cells/L |
| CD3+CD4+ cells | 1.185 | 0.3-2 x 10^9^ cells/L |
| CD3+CD8+ cells | 0.694 | 0.3-1.8 x 10^9^ cells/L |
| CD19+ cells | 0.279 | 0.2-1.6 x 10^9^ cells/L |
| CD16+CD56+ cells | 0.339 | 0.09 – 0.9 x 10^9^ cells/L |
| Anti-IFNα | Positive |  |
| Anti-IFNβ | Positive |  |
| Anti-IFNω | Positive |  |
| Anti-IL17A | Negative |  |
| Anti-IL17F | Positive |  |
| Anti-IL22 | Positive |  |
| Anti-GAD65 | 45.2 | <0.02 nmol/L |
| Anti-21-hydroxylase | 10 | <1 U/mL |
| Anti-thyroglobulin | <20 | 0-40 IU/mL |
| Anti-intrinsic factor | Negative |  |
| Anti-Ro | Negative |  |
| Anti-La | Negative |  |
| Anti-Sm | Negative |  |
| Anti-Jo-1 | Negative |  |
| Anti-Scl70 | Negative |  |
| Anti-VGKC | Negative |  |
| Anti-CASPR2 | Negative |  |
| Anti-LGI1 | Negative |  |
| **Anti-GlyR** | **Positive** |  |
| HLA typing | DRB1*01 DRB1*07 |  |

**Table S2.** **Clinical Manifestations of APECED and age of onset.**

| **Clinical manifestation** | **Age of onset (yrs)** |
| --- | --- |
| Urticarial eruption | 0.25 |
| Enamel hypoplasia | 5 |
| Hypoparathyroidism | 8 |
| Type 1 diabetes | 12 |
| Adrenal insufficiency | 12 |
| Alopecia areata | 13 |
| Testicular failure | 14 |
| PERM | 16 |

PERM, Progressive encephalomyelitis with rigidity and myoclonus.

**Figure S1. Brain MRI and CT Findings.**

**
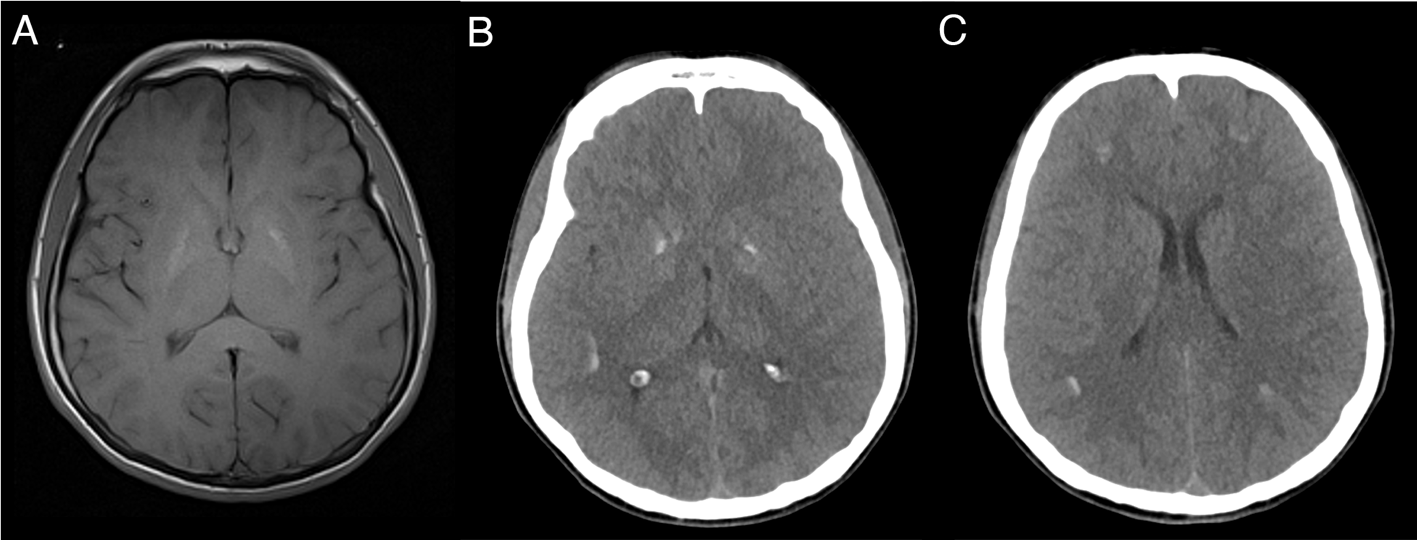
**

**Figure S1. Brain imaging findings at the time of NIH evaluation. A.** MRI brain T1 axial showing hyperintensities in the basal ganglia consistent with calcifications. **B.** Axial non-contrast head CT showing basal ganglia calcifications. **C.** subcortical white matter calcifications. CT, computed tomography; MRI, Magnetic Resonance Imaging.

**Abnormalities noted in the patient’s neurological physical exam**

- Non-fatigable symmetric bilateral ptosis

- Horizontal and vertical nystagmus (evoked by left gaze and upward gaze, respectively)

- Limited left eye abduction with esotropia

- Mildly slow tongue movements

- Neck stiffness (particularly of flexion and extension)

- Brisk deep reflexes throughout

- Difficulty with tandem gait without other evidence of ataxia of limbs or gait

- Hyperekplexia
